# Case report: Cardiac metastatic uterine intravenous leiomyomatosis excision with extracorporeal venous shunt under the guidance of 3-dimensional printing

**DOI:** 10.3389/fcvm.2023.1117227

**Published:** 2023-06-15

**Authors:** Hong Chen, Yu Mao, Hongtao Xie, Dejun Liu, Shun Zhang, Yongcang Tian, Jian Yang, Benjian Bai

**Affiliations:** ^1^ Department of Cardiovascular Surgery, The Second Affiliated Hospital of Shaanxi University of Chinese Medicine Xi’an New Area Central Hospital, Xi’an, China; ^2^ Department of Cardiovascular Surgery, Xijing Hospital, Air Force Medical University, Xi’an, China

**Keywords:** uterine leiomyomatosis, surgical treatment, extracorporeal venous shunt, 3-Dimensional printing, intravenous leiomyomatosis (IVL)

## Abstract

Intravenous leiomyomatosis (IVL) is relatively rare, and the incidence of cardiac IVL is even lower. The case report introduces a 48-year-old woman with two episodes of syncope in 2021. Echocardiography showed a cord-like mass in the inferior vena cava (IVC), right atrium (RA), right ventricle (RV) and pulmonary artery. Computed tomography venography and magnetic resonance imaging showed strips in RA, RV, IVC, right common iliac vein, and internal iliac vein, as well as a round-like mass in the right uterine adnexa. Combined with the patient's prior surgical history and rare anatomical structures, surgeons used cardiovascular 3-dimensional (3D) printing technology to create patient-specific preoperative 3D printed model. The model could help surgeons to visually and accurately understand the size of IVL and its relationship to adjacent tissues. Finally, surgeons successfully performed a concurrent transabdominal resection of cardiac metastatic IVL and adnexal hysterectomy with off-cardiopulmonary bypass. Preoperative evaluation and guidance of 3D printing may play a critical role to ensure this surgery for the patient with rare anatomical structures and high surgical risk.

**Clinical Trial Registration:** [ClinicalTrials.gov], Protocol Registration System [NCT02917980].

## Introduction

Intravenous leiomyomatosis (IVL) is a distinct uterine leiomyomatosis, which is a special type of benign tumor with adverse biological behaviors and mainly originates from uterine fibroids. IVL is rare in clinic, and tumor invasion to the right cardiac system is even rarer (1). In 1975, Norris et al. made a comprehensive and systematic analysis of the clinical and characteristics of IVL (2). The source of IVL is usually pelvic organs, and the tumor is diffused by smooth muscle cells along the vascular wall of renal vein or pelvic vein, and even extends into the inferior vena cava (IVC) and then into the right cardiac system (RCS) (3). With insidious onset and lack of clinical characteristics, IVL is easy to be misdiagnosed and missed (4). Most patients present with syndromes of RCS, such as shortness of breath, paroxysmal syncope. IVL entering RCS may be misdiagnosed as other tumors or thrombus due to space occupying lesions in the right atrium (RA). Although histologically benign, IVL may lead to circulatory failure and death if left untreated (5). Radical excision is the only effective treatment at present (6). However, due to the complex anatomical structures of patients with common pathological conditions and the high surgical risk, the surgery is a major challenge for cardiovascular surgeons. In this case report, we introduced the application of cardiovascular 3-dimensional (3D) printing technology to provide auxiliary guidance for preoperative evaluation, aiming to provide help for the clinical diagnosis and treatment of such patients.

## Timeline

**Table:** 

Time	Events
7 months ago	The first time of syncope
1 day ago	The second time of syncope
Day 0	Hospitalization
Preoperative day 1	Assessment of computed tomography, echocardiography and magnetic resonance imaging
Preoperative day 2	The 3D-printed model was reconstructed and the surgical strategy was then formulated
Operative day	Cardiac metastatic uterine intravenous leiomyomatosis excision with extracorporeal venous shunt
Postoperative day 8	The patient was in stable condition, discharged without complications

## Preoperative baseline characteristics

**Figure 1:**
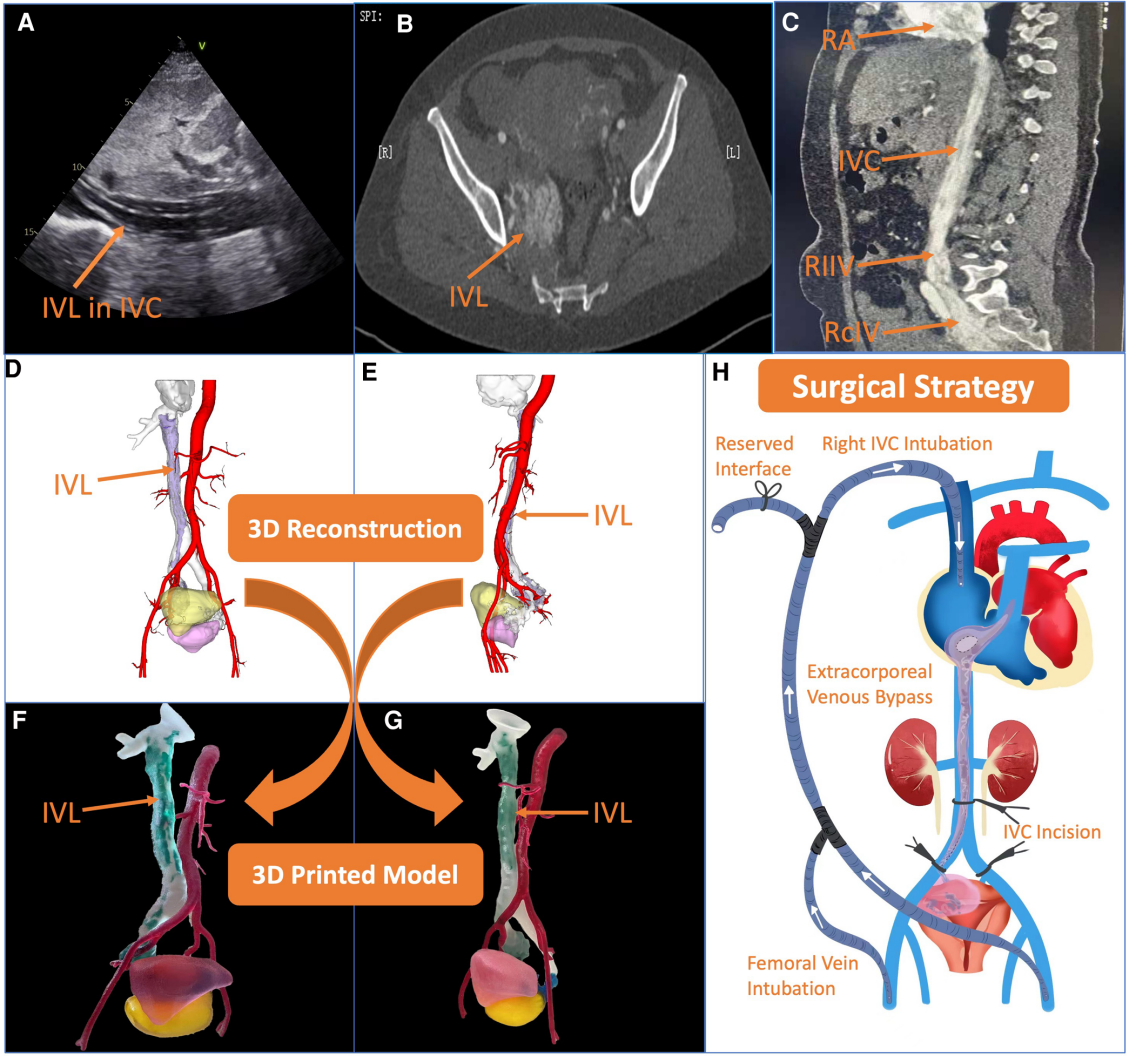
Preoperative assessment and surgical strategy formulation. (**A–C**) Echocardiography, computed tomography and magnetic resonance imaging showed intravenous leiomyomatosis (IVL) in different anatomical sites clearly. (**D,E**) 3-dimensional (3D) reconstruction using Materialise 3-matic (Leven, Belgium) software in the lateral view and front view, respectively. The red part is abdominal aorta, the transparent part is inferior vena cava (IVC), the purple part is IVL, the yellow part is bladder, and the fuchsia part is uterus. (**F,G**) The 3D printed model in the lateral view and front view, respectively. The red part is abdominal aorta, the transparent part is IVC, the green part is cardiac metastatic IVL, the blue part is uterine IVL, the pink part is bladder, and the yellow part is uterus. (**H**) After comprehensive evaluation, the surgical strategy was decided, transabdominal cardiac metastatic IVL and adnexal hysterectomy were proposed at the same time under off-cardiopulmonary bypass with the establishment of an external venous bypass (bilateral femoral vein-superior vena cava bypass) to maintain circulation.

The 48-year-old woman was suffered from shortness of breath for more than one year. In 2021, the patient had aggravated chest tightness and shortness of breath after activities, and two episodes of syncope was occurred. Echocardiography revealed a cord-like mass in IVC, RA, right ventricle (RV) and pulmonary artery (PA) that moved with cardiac contraction (Figure 1A). Computed tomography venography (CTV) showed strips in RA, RV, IVC, right common iliac vein (RcIV) and internal iliac vein (IIV). The lesions of IVC and RcIV showed irregular simoid-like changes, and the lesions of distal right internal iliac vein (RIIV) showed a 3.0 cm × 5.1 cm mass. Multiple round weak enhancement shadows were seen in the uterus. A 30 mm lesion could be seen in the right accessory area (Figure 1B). Magnetic resonance imaging (MRI) results were consistent with CTV (Figure 1C). Combined with the patient's hysteromyectomy history in 2020, the patient was finally diagnosed with cardiac metastatic uterine IVL and uterine fibroids.

## 3D printed model and surgical plan formulation

The Digital Imaging and Communication of Medicine format of the patient's CTV data was imported into Materialise Mimics version 21.0 (Leven, Belgium) software, and the 3D reconstructed model was segmented by the threshold segmentation function. In Materialise 3-matic (Leven, Belgium) software, The 3D model was processed digitally, such as shell extraction, cutting, smoothing and repair, and the tissue structure and the shape of the false cavity were completely restored (Figures 1D,E). The Standard Tessellation Language (STL) file of the 3D reconstructed model was exported to Stratasys Polyjet 850 multi-material full color 3D printer. Different tissues were edited and printed with soft, hard and colored materials to obtain a 3D printed model of the patient.

According to the 3D printed model, we could intuitively and fully understand the anatomical morphology of the right uterine adnexa, RIIV, RcIV, IVC, RA, RV and PA (Figures 1F,G). By intuitively and accurately understanding the size of the tumor and the relationship between the tumor and the adjacent tissues, surgeons could more effectively plan the operation and formulate the surgical plan. From the model, surgeons could be observed that there was no obvious adhesion between tumor and IVC clearly. In case, we also reserved an interface on *extracorporeal venous shunt* pipe, which simulated on the 3D printed model before the surgery. The *extracorporeal venous shunt* was designed from bilateral femoral veins to right internal jugular vein. When IVC was blocked, the lower limb blood could flow into RA via the superior vena cava through the bypass, so as to ensure intraoperative hemodynamic stability. After multidisciplinary preoperative discussions, transabdominal cardiac metastatic IVL and adnexal hysterectomy were proposed at the same time under off-cardiopulmonary bypass with the establishment of an extracorporeal venous bypass (bilateral femoral vein-superior vena cava bypass) to maintain circulation (Figure 1H).

## Surgical steps and perioperative follow-up outcomes

**Figure 2:**
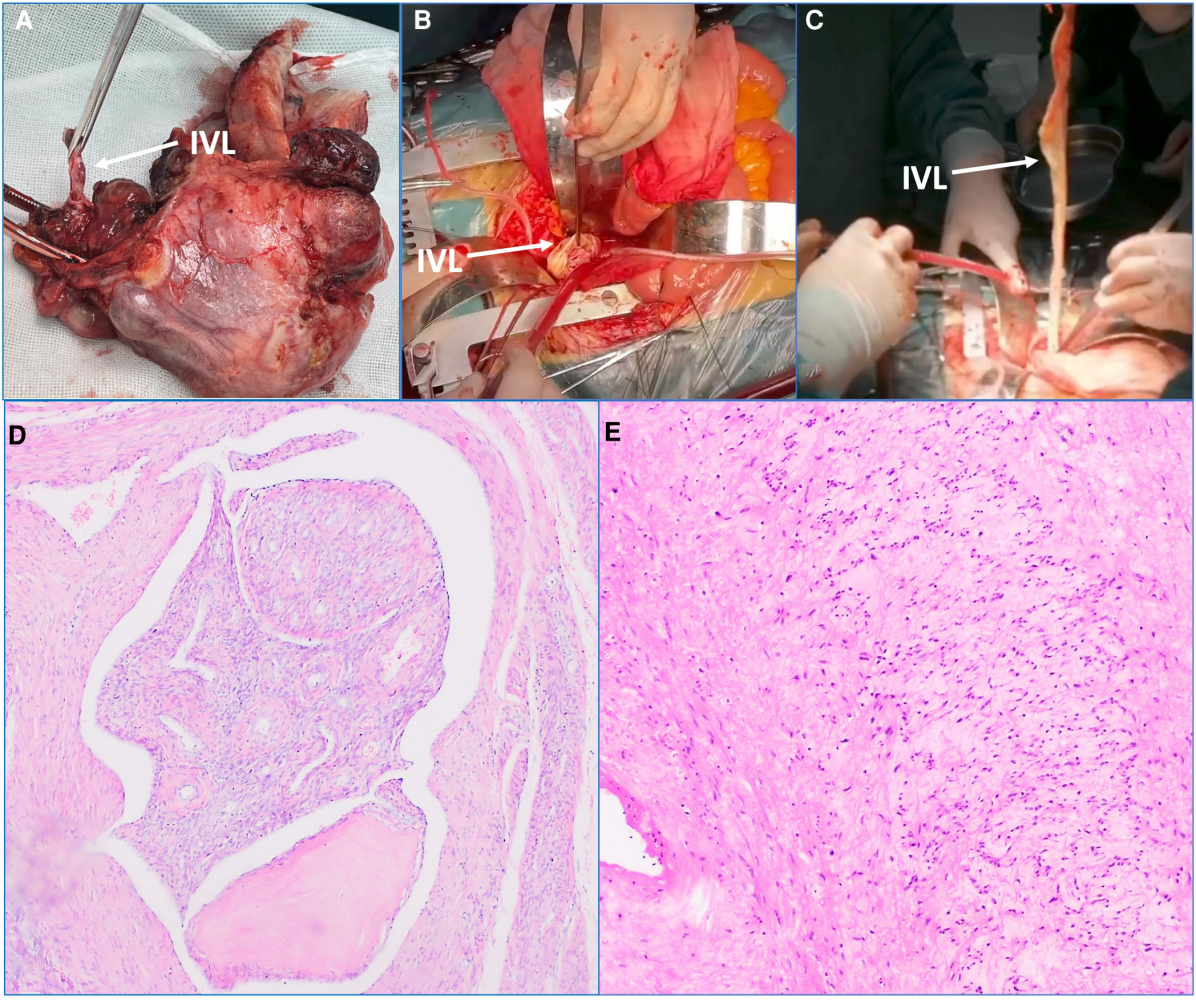
Main intraoperative steps and postoperative pathological examination results. (**A**) The uterus and the surrounding tissues were excised, and the arrow heads to the intravenous leiomyomatosis (IVL). (**B,C**) The tubula-like IVL was found in inferior vena cava (IVC). The arrow heads to IVL. (**D**) The excised tissues in uterus were sent to the pathological examination. The tumor cells are generated in the lobed shape in the vascular lumen. Immunohistochemical results: CD34 (+), Desmin (+), SMA (+), calponin (+), CD31 (+). This result was consistent with the preoperative diagnosis. (**E**) The excised tissues in IVL were sent to the pathological examination. Immunohistochemical results: CD34 (+), Desmin (+), SMA (+), calponin (+), CD31 (+). This result was consistent with the preoperative diagnosis.

First of all, after the patient underwent general anesthesia, the gynecologist took the patient's lithotomy position to excise the uterus and the surrounding tissues, and the part of excised tissues were sent to the pathological examination (Figure 2A). Then, the cardiovascular surgeon placed the patient in supine position, and the anesthesiologist injected heparin intravenously at the rate of 1 mg/kg, which maintaining activated clotting time (ACT) >400 s. Then, surgeons inserted an 18 Fr core cannula from the right internal jugular vein puncturing into RA, and separated the bilateral femoral arteries and femoral veins, and then inserted another 18 Fr core cannula into the bilateral femoral veins, established the blood flow circuit from the bilateral femoral veins to RA through the right internal jugular vein.

There were no significant changes in heart rate and blood pressure after the establishment of extracorporeal venous bypass circulation. Subsequently, IVC was longitudinally incised to free IVC and bilateral cIVs. The occlusion was completed 2 cm below the renal vein and cIVs, respectively. IVC was cut longitudinally between the blocking bands and extended to the RcIV. The tumor was caught by angled clamps, and the blocking band near IVC was loosened to pull out the tumor in the heart cavity (Figures 2B,C). Then, the blocking bands of IVC and RcIV were blocked, respectively; and the residual 45 cm tumor was cut off. The excised specimens were sent for the pathological examination, and finally the venous incision was sutured.

In addition, the pelvic lesions, IVC and the non-cohesive tumor in the heart cavity were excised at the same time. The intraoperative and postoperative hemodynamics were observed stable, and the central venous pressure decreased from 17 cmH_2_O before surgery to 8 cmH_2_O after surgery immediately. Postoperative pathological examinations verified IVL and uterine fibroids (Figures 2D,E). The endotracheal tube was removed 8 h later, the patient recovered quite well and was discharged successfully 10 days later.

## Discussion

IVL is mainly derived from uterine smooth muscle or pelvic vascular smooth muscle tissues. Tumors can extend to pelvic veins, iliac veins or renal veins, gradually involve IVC, and extend into RA, RV or even PA (7, 8). The IVL extends along the para-ventral vein to IVC and invades RA, RV and evenPA, causing syncope. In addition, IVL entering the right cardiac system is easily misdiagnosed as thrombus due to atrial space occupying lesions. Although histologically benign, untreated IVL into the heart may lead to circulatory failure. The growth process of IVL is similar to that of malignant tumors and has the characteristic of aggressive growth. Above all, multimodal imaging should be taken, especially for female patients with space occupying lesions in RCS. Studies have shown that 30%–80% of patients with IVL *in utero* had extrauterine involvement, of which 10%–30% patients occurred in the heart (9). However, the cause remains unclear. However, studies have found that the level of estrogen in IVL patients is relatively high, and the level of estrogen receptor in smooth muscle cells of IVL is significantly higher than that in smooth muscle cells of normal uterine/uterine fibroids, suggesting that high estrogen level is related to the occurrence and development of IVL (10). IVL has been reported to occur in women aged 28–80 years (median age 45 years), most of whom are perimenopausal (11). However, antiestrogenic therapy did not significantly improve the prognosis of IVL (12).

In principle, once IVL is found, operation should be performed as soon as possible. The surgical strategies include concurrent surgery and staged surgery, which tend to be multi-disciplinary collaboration for concurrent radical surgery (13–17). In common, midline xifo-pubic laparotomy, allowing exposure of the entire inferior vena cava are used to complete exploration of the cardiac atrium and if necessary right ventricle and pulmonary artery (18–21). Due to the staged operation, the intracardiac and proximal IVC tumors are removed by thoracotomy under extracorporeal circulation, and then the residual tumors are removed by transabdominal resection in the next stage. Two operations increased the physiological and psychological burden of the patients. Because IVL is rare, the surgical treatment is highly individualized. The selection of surgical strategies varies according to the location of tumor. It is worth noting that the patients with clear preoperative diagnosis were all operated under extracorporeal circulation, and the patients did not have deep hypothermia and stopped circulation, which created a good condition for the postoperative recovery of the patients. Therefore, the accurate preoperative diagnosis, the determination of the invasion scope and origin of the tumor are of great significance for the surgical strategy and prognosis. At the same time, the onset of IVL is relatively insidious, and the early clinical manifestations lack specificity. Echocardiography is a common auxiliary examination for understanding pelvic mass, but often shows uterine enlargement and multiple nodules, which is similar to that of common uterine fibroids. CT and MRI are also difficult to diagnose tumors before invading blood vessels or when the anatomical morphology is abnormal. Although pathological examination is the golden standard of diagnosis, it is not suitable for preoperative evaluation. In addition, 2-dimensional images are not easy to distinguish the relationship between early uterine masses and intravascular masses, so innovative preoperative evaluation methods are urgently needed for comprehensive evaluation. With the continuous development of cardiovascular 3D printing technology, surgeons use 3D printed models for preoperative simulation evaluation, which plays an important auxiliary and guiding role in the accurate formulation of surgical plans and the improvement of the success rate of surgery. Preoperative CT data of the patient were evaluated in detail, and 3D printed model of the lesion and adjacent tissues of the patient were carried out. In addition, the 3D printed model was used to accurately understand the specific anatomical structures, the size of the tumor and the relationship between the tumor and adjacent tissues, so as the surgical program and strategy were reasonably planned (22). In this case, the tumor was extracted directly through IVC incision. The preoperative 3D printed model could be showed no obvious adhesion between tumor and IVC clearly. In case, we also reserved an interface on *extracorporeal venous shunt* pipe, which simulated on the 3D printed model before the operation; then further completed the operation through RA approach and ensure the safety of procedures, which is the highlight of this operation.

In addition, it is recognized that cardiopulmonary bypass assisted cardiac arrest is performed to remove the tumor through thoracotomy or thoracoabdominal combined incision (23). Although this method is relatively safe, it is characterized by great trauma and more complications. In order to reduce trauma and complications, we designed a set of *extracorporeal venous shunt*, which circulates the lower limb venous blood through the right internal jugular vein to RA through pipelines. When blocking IVC, the intraoperative hemodynamic is maintained stable, and the local hemodynamic status may be achieved to facilitate the treatment of lesions. In particular, an interface was reserved on the venous bypass pipeline that can be connected with the extracorporeal circulation venous pipeline, so that the extracorporeal circulation of femoral arteriovenous return can be established during the operation. The advantages of this method are as follows: (a) Extracorporeal venous bypass shunt did not require thoracotomy for vena cava intubation, which avoided the difficulty of intubation in IVC due to tumor blockage during the establishment of extracorporeal circulation, and also reduced the impact of extracorporeal circulation on the patient. (b) A relatively bloodless area may be provided by blocking IVC, which was convenient for removing lesions in the heart, and could also treat the lesions in the pelvic cavity at the same time, avoiding staged surgeries and the influence of thoracotomy or combined thoracoabdominal incision. (c) IVL consists of well-differentiated smooth muscle cells covered with smooth endothelium, which can be completed tumor resection. However, in a small number of patients, the tumor will be adhered to the heart or IVC, or massive bleeding occurs during the operation. Femoral artery intubation could also be performed and the reserved orifice of the venous bypass could be connected to immediately establish extracorporeal circulation to complete the operation to ensure the safety. (d) The method is simple and easy to perform, and is suitable for other diseases causing IVC obstruction. In addition, the majority of IVL was solid structures, and some had sac-like changes. However, the tumor of this patient had a tubular structure, and the wall was arterial smooth muscle with uniform thickness and elasticity, rather than uterine and venous smooth muscle, which had not been reported in the previous studies.

In conclusion, IVL is clinically rare and has invasive growth potential, so successful surgical treatment of IVL is highly dependent on complete resection of the tumor. Therefore, it is important to understand its clinical features and to conduct adequate preoperative evaluations under the guidance of 3D printing. In this case report, we introduce the application of cardiovascular 3D printing technology, which plays an innovative and important auxiliary guiding role in improving the quality of preoperative evaluation, so as to help surgeons establish extracorporeal venous bypass (bilateral femoral vena-superior vena cava bypass) to maintain circulation. In addition, cardiac metastatic IVL and adnexal hysterectomy could be performed at the same time under off-cardiopulmonary bypass.

## Data Availability

The original contributions presented in the study are included in the article, further inquiries can be directed to the corresponding author/s.
